# An Exceptionally Preserved Transitional Lungfish from the Lower Permian of Nebraska, USA, and the Origin of Modern Lungfishes

**DOI:** 10.1371/journal.pone.0108542

**Published:** 2014-09-29

**Authors:** Jason D. Pardo, Adam K. Huttenlocker, Bryan J. Small

**Affiliations:** 1 Department of Biological Sciences, University of Calgary, Calgary, Alberta, Canada; 2 Department of Biology, University of Utah, and Natural History Museum of Utah, Salt Lake City, Utah, United States of America; 3 Museum of Texas Tech University, Lubbock, Texas, United States of America; 4 Rocky Mountain Dinosaur Resource Center, Woodland Park, Colorado, United States of America; Penn State University, United States of America

## Abstract

Complete, exceptionally-preserved skulls of the Permian lungfish *Persephonichthys chthonica* gen. et sp. nov. are described. *Persephonichthys chthonica* is unique among post-Devonian lungfishes in preserving portions of the neurocranium, permitting description of the braincase of a stem-ceratodontiform for the first time. The completeness of *P. chthonica* permits robust phylogenetic analysis of the relationships of the extant lungfish lineage within the Devonian lungfish diversification for the first time. New analyses of the relationships of this new species within two published matrices using both maximum parsimony and Bayesian inference robustly place *P. chthonica* and modern lungfishes within dipterid-grade dipnoans rather than within a clade containing Late Devonian ‘phaneropleurids’ and common Late Paleozoic lungfishes such as *Sagenodus*. Monophyly of post-Devonian lungfishes is not supported and the Carboniferous-Permian taxon *Sagenodus* is found to be incidental to the origins of modern lungfishes, suggesting widespread convergence in Late Paleozoic lungfishes. Morphology of the skull, hyoid arch, and pectoral girdle suggests a deviation in feeding mechanics from that of Devonian lungfishes towards the more dynamic gape cycle and more effective buccal pumping seen in modern lungfishes. Similar anatomy observed previously in *‘Rhinodipterus’ kimberyensis* likely represents an intermediate state between the strict durophagy observed in most Devonian lungfishes and the more dynamic buccal pump seen in *Persephonichthys* and modern lungfishes, rather than adaptation to air-breathing exclusively.

## Introduction

Modern ceratodontiform lungfishes are the closest living relatives to tetrapods. Though relatively species poor today, the lungfish stem-group (Dipnoi) was well represented in the Late Paleozoic, originating in the Early Devonian [1] and exhibiting an apparently rapid adaptive radiation [2]. In some cases, these adaptations mirrored those found in early tetrapod evolution, including loss of the intracranial joint, reduction of hypermineralized dermal tissues, loss of a static bony connection between the pectoral girdle and skull, and the parallel evolution of depressor mandibulae musculature [3], [4]. Despite these similarities, significant differences exist between the diversifications of lungfishes and tetrapodomorphs. Lungfishes exhibited important early innovation in their dentition, losing the marginal tooth-bearing bones entirely and instead extrapolating the palatal dentition into various forms of tooth plate or denticle field [5], a characteristic that fostered high diversity of durophagous lungfishes in marine reef environments up to the Famennian-Frasnian boundary. Canalization of the dentition within the Late Devonian [6] and subsequent turnover of forms with alternate dental arcades [5], [7] coincided with a change in mode of lungfish evolution. Consequently, post-Devonian lungfishes demonstrate remarkably conserved dentitions [6] in spite of wide disparity in dermal cranial anatomy [8] and perhaps aspects of their endocranial morphology. This conservative dental morphology has fostered a more general observation first made by Darwin [9] and subsequently extrapolated by a number of other workers [2], [10] that post-Devonian lungfishes are evolutionarily conservative to a singular degree, with little morphological evolution or change in niche since the Kellwasser Extinction Event.

Although lungfishes present an important comparison with early tetrapod evolution, as well as an interesting evolutionary problem in their own right, our understanding of the history of this group, and specifically the transition between the initial Devonian diversification to the lungfish crown itself, remains poorly understood. This is likely a result of the quality of the fossil record of late Paleozoic lungfishes; current understanding of the anatomy of Carboniferous and Permian lungfishes is restricted to tooth plate morphology and superficial anatomy of the dermal skeleton. This has presented a persistent challenge for workers interested in reconstructing lungfish phylogeny, and has led to fundamentally different interpretations of homology, phylogeny, and evolution [5], [11]–[13]. For Devonian lungfishes, this has been ameliorated to some degree by the development of a large character matrix comprised primarily of neurocranial characters [13] and subsequently expanded to include additional published characters [14]. This dataset cannot be extended to late Paleozoic lungfishes, however, as no neurocranium has been described for late Paleozoic lungfishes until now. The absence of neurocranial data for fossil lungfishes from the Carboniferous onwards has been attributed to a wholesale reduction of endochondral ossification within the braincase of post-Devonian lungfishes, as well as some Late Devonian forms, such as *Scaumenacia curta* [15], but it has remained unclear whether this represents a real biological signal, or simply a taphonomic bias against three-dimensional preservation of delicate structures in latest Devonian and post-Devonian lungfishes.

Here we report on exceptionally-preserved skulls of a new genus and species of lungfish from the earliest Permian Eskridge Formation of Nebraska, USA. These skulls were previously reported [16]–[19] as a gnathorhizid, based on the arrangement of cranial bones and the highly sectorial tooth plates, and as cf. *Monongahela* based on the presence of an expanded coronoid eminence, but have never been formally described. High resolution x-ray computed tomography (HRXCT) reveals intricate details of the skull, including the first neurocranium from a fossil post-Devonian lungfish. These exceptionally-preserved skulls present a mosaic of characters found in both archaic Devonian forms and derived members of the dipnoan crown, and represent a singular intermediate form in an otherwise poorly-represented evolutionary transition.

## Methods

### Specimens studied

All material studied here is permanently reposited in the vertebrate paleontology collections of the University of Nebraska State Museum (UNSM) in Lincoln, NE, USA. No permits were required for the described study, which complied with all relevant regulations.

### HRXCT

Two skulls (UNSM 32104 and UNSM 32108) were submitted to HRXCT scanning at the University of Calgary. Complete scans of both skulls were conducted using a Skyscan1173 µCT machine (Kontich, Belgium) at 100 kV and 60 µA with an aluminum filter and a voxel size of 38.9 µm. A high resolution scan of the ethmoid region of UNSM 32108 was produced using the same machine at energy levels of 75 kV and 95 µA with no filter and a voxel size of 11.36 µm. A high resolution scan of the occipital region of UNSM 32104 was produced at energy levels of 80 kV and 80 µA with no filter and a voxel size of 13.49 µm. Scans were reconstructed as slice stacks in nrecon version 1.6.6.0 (Skyscan, 2011). Stacks were segmented and rendered as 3D models in Amira 5.4.0 (Visage Imaging, San Diego, CA); whole-scan volume renders were produced using the VolRen module, and individual elements were segmented using the LabelField module and rendered using the SurfaceGen module.

### Phylogenetic analysis

We assessed the relationships of the new lungfish with Devonian lungfish species, and the impact of this new species on resolution of lungfish phylogeny, using the matrix of Qiao & Zhu [14], which incorporates the characters of Friedman [13] within a comprehensive sampling of skull roof, dental, and postcranial characters drawn primarily from Schultze [1] and Ahlberg et al., [5] (Appendix S1). We additionally coded the new taxon into the braincase-only matrix of Friedman [13] as modified by Clement [20] (Appendix S2) to test whether the relationships of *Persephonichthys chthonica* within Devonian lungfishes remained robust to removal of homoplastic skull roof characters. For the neurocranium-only analysis, 70 neurocranial characters were coded for 28 taxa. For the comprehensive matrix, 150 characters were coded for 38 taxa, including 34 taxa previously coded by Qiao and Zhu [14], *Persephonichthys chthonica*, *Neoceratodus forsteri*, *Sagenodus copeanus* [21] and Lepidosireniformes as a composite taxon. The gnathorhizid *Gnathorhiza* was not included in this analysis, as a revision of the morphology and taxonomy of this genus is necessary. All characters were left unmodified for the neurocranial matrix. Character 9 (Paired E bones) was modified in the comprehensive matrix to include an additional state: unpaired, which was coded for *Orlovichthys limnatis*, *Persephonichthys chthonica*, *Neoceratodus forsteri*, and Lepidosireniformes. Both matrices were analyzed using maximum parsimony and maximum posterior probability as objective criteria. Maximum parsimony analyses were conducted in PAUP*4.0b [22] and maximum posterior probability was conducted in MRBAYES 3.0 (Ronquist etal., 2011) using the default Mk1 model for morphological character evolution. Support values for maximum parsimony analysis were calculated via bootstrap resampling with replacement of 132 characters for 1000 replications for each matrix.

### Nomenclatural Acts

The electronic edition of this article conforms to the requirements of the amended International Code of Zoological Nomenclature, and hence the new names contained herein are available under that Code from the electronic edition of this article. This published work and the nomenclatural acts it contains have been registered in ZooBank, the online registration system for the ICZN. The ZooBank LSIDs (Life Science Identifiers) can be resolved and the associated information viewed through any standard web browser by appending the LSID to the prefix “http://zoobank.org/”. The LSID for this publication is: urn:lsid:zoobank.org:pub:9BD2D360-C8D0-4130-A211-2466F30537B1. The electronic edition of this work was published in a journal with an ISSN, and has been archived and is available from the following digital repositories: PubMed Central, LOCKSS.

## Results

### Systematic Paleontology

Osteichthyes Huxley 1880

Dipnoi Müller 1845

***Persephonichthys chthonica*** gen. et **sp. nov.** Pardo, Huttenlocker, and Small urn:lsid:zoobank.org:act:B8A4A5BC-9F3D-4052-9573-3C1010FE9270

(Figures 1–5)

**Figure 1:**
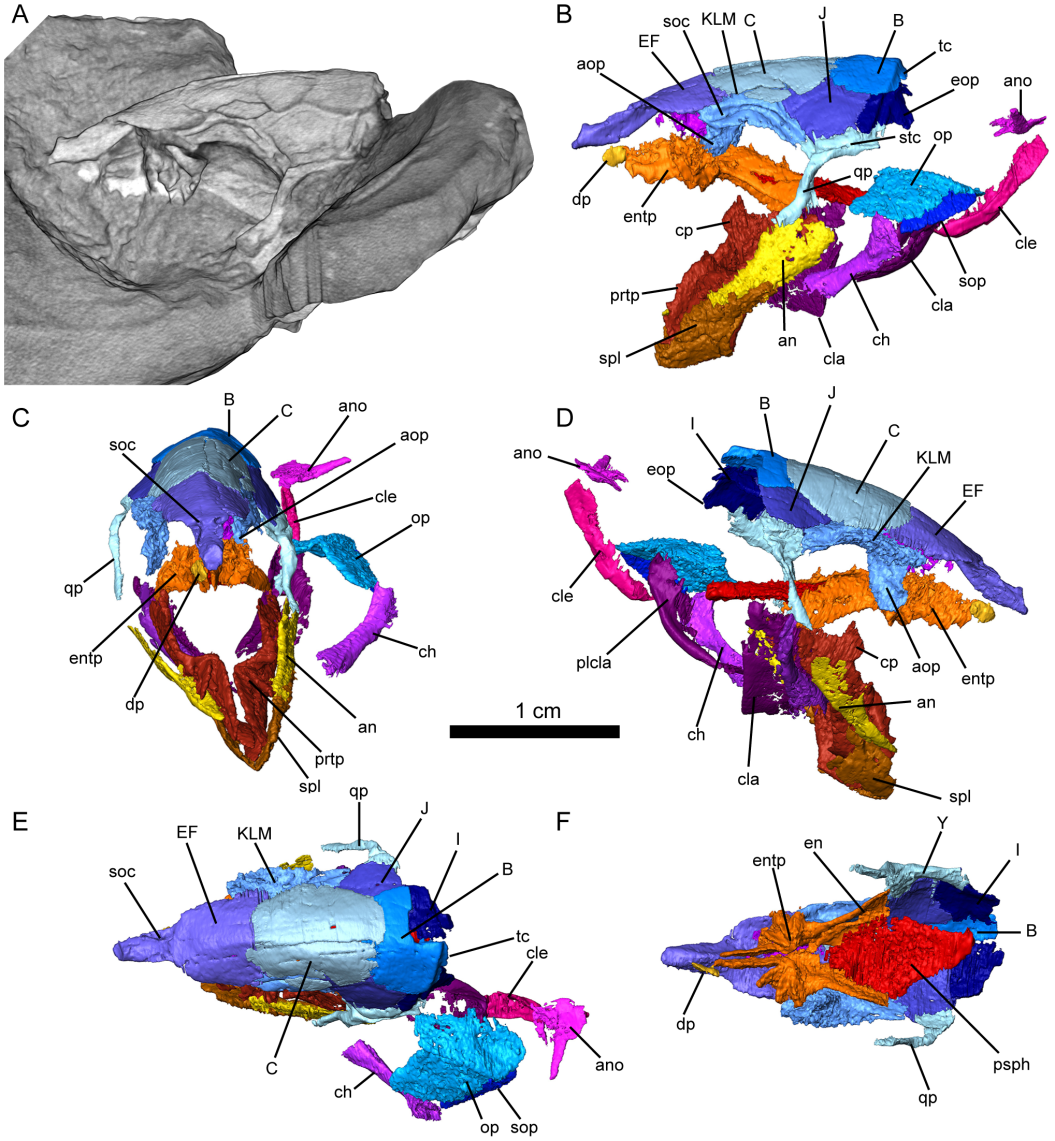
*Persephonichthys chthonica*, holotype, UNSM 32108. **A**, volume render of micro-CT scan of UNSM 32108, left lateral view; **B–F**, segmented micro-CT scan of UNSM 32108; **B**, left lateral view; **C**, anterior view; **D**, right lateral view; **E**, dorsal view; **F**, palatal view, with lower jaw, branchial, and pectoral elements removed. Scale bar equals 10 mm. Abbreviations: B, C, EF, I, J, KLM, Y, bones of the skull; an, angular; ano, anocleithrum; aop, antorbital process of KLM; ch, ceratohyal; cla, clavicle; cle, cleithrum; cp, coronoid process of prearticular; dp, dermopalatine; en, entopterygoid; entp, entopterygoid tooth plate; eop, exoccipital process of bone I; op, operculum; plcla, posterior lamina of clavicle; prtp, prearticular tooth plate; psph, parasphenoid; qp, quadrate process of bone Y; soc, supraorbital canal; sop, suboperculum; spl, splenial; stc, supratemporal canal; tc, temporal commissure.

**Figure 2:**
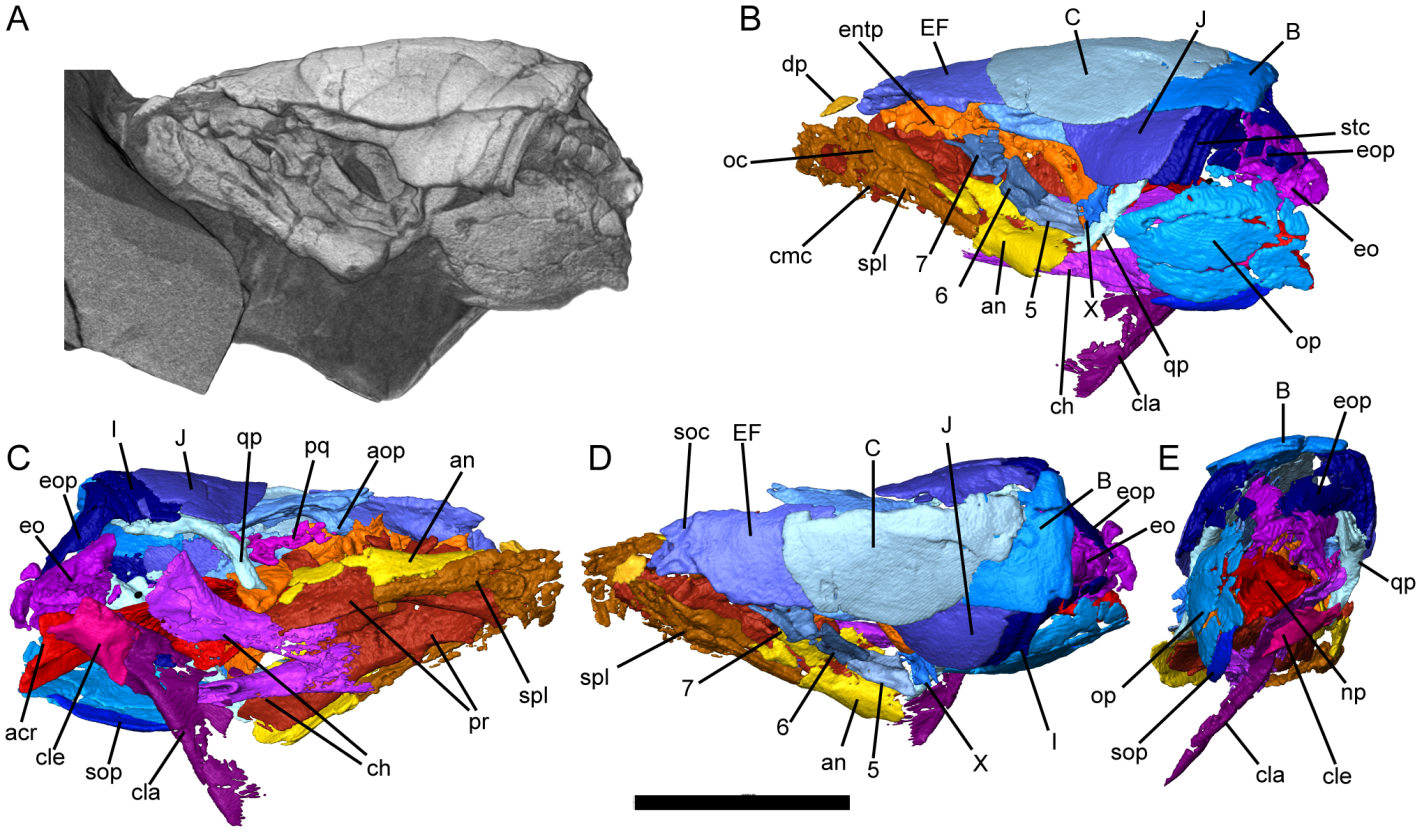
*Persephonichthys chthonica*, referred specimen, UNSM 32104. **A**, volume render of micro-CT scan of UNSM 32104, left lateral view; **B–E**, segmented micro-CT scan of UNSM 32104; **B**, left lateral view; **C**, right ventrolateral view; **D**, dorsal view; **E**, occipital view. Scale bar equals 10 mm. Abbreviations: B, C, EF, I, J, KLM, Y, bones of the skull; 5, 6, 7, 8, circumorbital bones; acr, articulation of the cranial rib; an, angular; aop, antorbital process of bone KLM; ch, ceratohyal; cmc, commissural branch of the mandibular canal; cla, clavicle; cle, cleithrum; dp, dermopalatine; entp, entopterygoid tooth plate; eo, exoccipital; eop, exoccipital process of bone I; np, notochordal pit; oc, oral canal; op, operculum; pr, prearticular; qp, quadrate process of bone Y; soc, supraorbital canal; sop, suboperculum; spl, splenial; stc, supratemporal canal.

**Figure 3:**
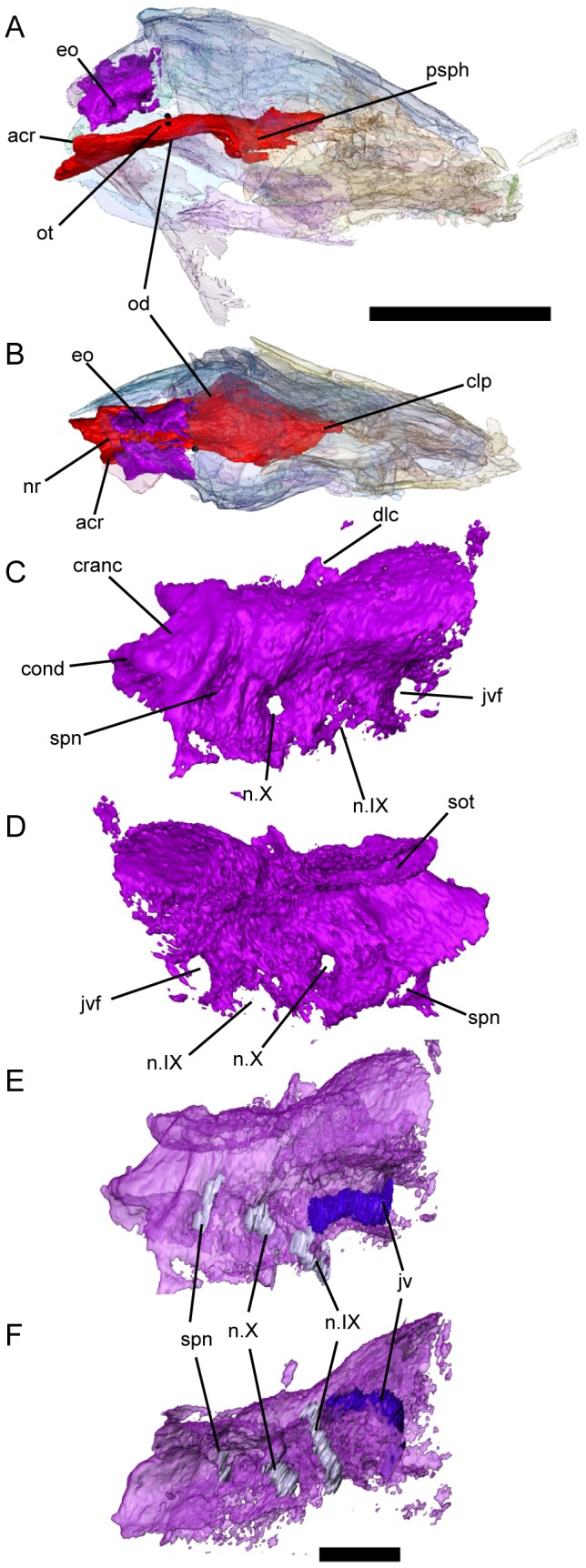
Occipital braincase elements of *Persephonichthys chthonica*, referred specimen, UNSM 32104. **A**, right lateral view of skull, showing position of the occipital bones; **B**, dorsal view of skull, showing position of the occipital bones; **C**, right exoccipital bone, lateral view; **D**, right exoccipital bone, medial view; **E**, endosteal structures of the right exoccipital bone, lateral view; **F**, endosteal structures of the right exoccipital bone, ventral view. Scale bar: **A–B**, equals 10 mm; **C–F**, equals 1 mm. Abbreviations: acr, articulation of the cranial rib; clp, cultriform process of parasphenoid; cranc, cranial centrum; dlc, dorsolateral crista; eo, exoccipital; jv, jugular vein; jvf, jugular vein foramen; n.IX, glossopharyngeal nerve; n.X, vagus nerve; nr, notochordal recess; od, otic depression; ot, saccular otolith; psph, parasphenoid; spn, spinal nerve.

**Figure 4:**
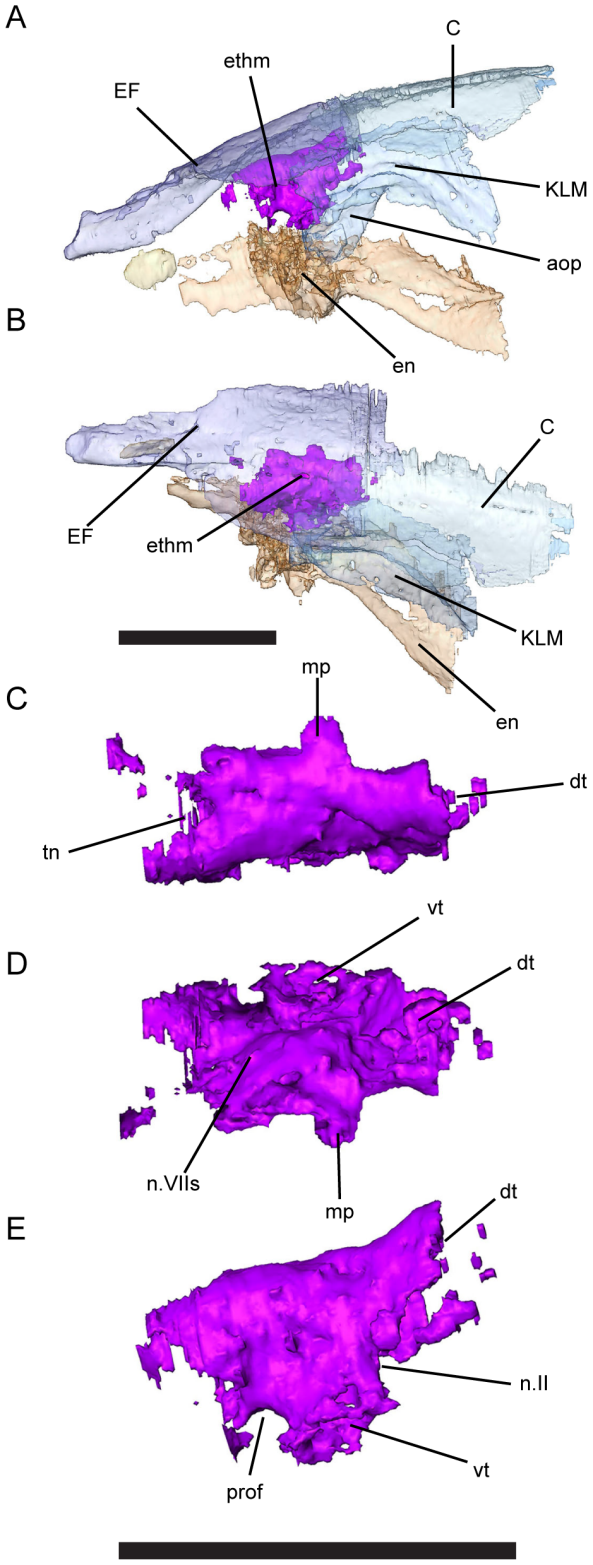
Ethmoid element of *Persephonichthys chthonica*, holotype, UNSM 32108. **A**, left lateral view of skull, showing position of the ethmoid ossification; **B**, dorsal view of skull, showing position of the ethmoid ossification; **C**, ethmoid, dorsal view; **D**, ethmoid, ventral view; **E**, ethmoid, lateral view. Scale bar equals 5 mm. Abbreviations: C, EF, KLM, bones of the skull; aop, antorbital process of bone KLM; dt, dorsal trabeculum; en, entopterygoid; ethm, ossification of ethmoid; mp, median process; n.II; optic nerve; n.VIIs, superficialis branch of facial nerve; prof, profundus nerve; tn, tectum nasalis; vt, ventral trabeculum.

**Figure 5:**
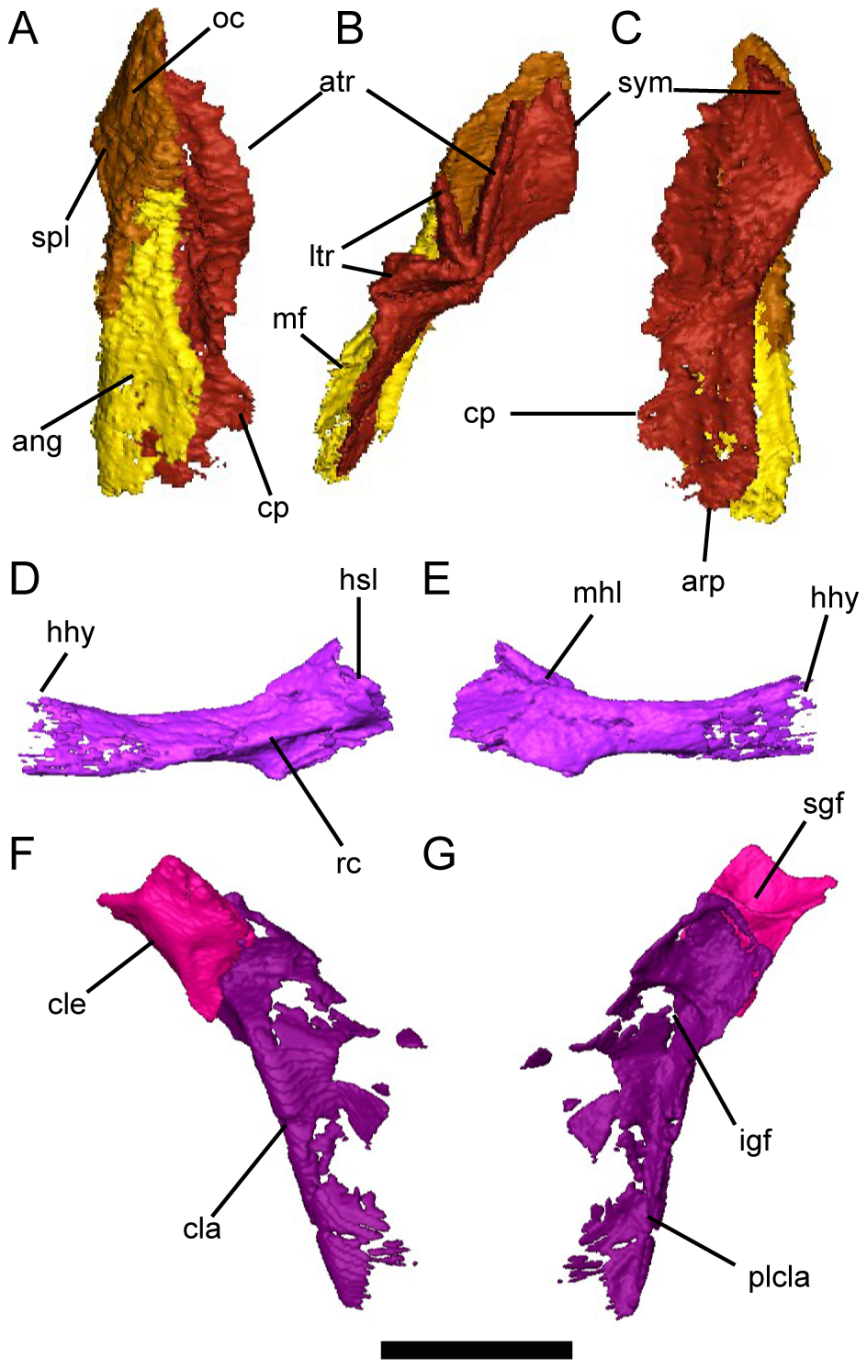
Lower jaw, branchial, and pectoral skeleton of *Persephonichthys chthonica*. **A**, left lower jaw of UNSM 32108, left lateral view; **B**, left lower jaw of UNSM 32108, occlusal view; **C**, left lower jaw of UNSM 32108, medial view; **D**, left ceratohyal of UNSM 32104, lateral view; **E**, left ceratohyal of UNSM 32104, medial view; **F**, partial right pectoral girdle of UNSM 32104, lateral view; **G**, partial right pectoral girdle of UNSM 32104, medial view. Scale bar equals 5 mm. Abbreviations: an, angular; arp, articular process of prearticular; atr, anterior tooth row of prearticular tooth plate; cla, clavicle; cle, cleithrum; cp, coronoid process of prearticular; hy, articulation with hypohyal; hsl, insertion of hyosuspensory ligament; igf, infraglenoid fossa; ltr, lateral tooth rows of prearticular tooth plates; mf, mandibular fossa; mhl, mandibulohyoid ligament; oc, oral canal; plcla, posterior lamina of clavicle; rc, insertion of rectus cervicus muscle; sgf, supraglenoid fossa; spl, splenial; sym, symphysis.

#### Etymology

From *Persephone*, a Greek goddess whose annual descent and re-emergence from the underworld is associated with the change of seasons, in reference to the seasonal aestivation burrows that fossils of this species are found in, and *ichthys*, meaning fish. Specific name meaning “from underground” or “from the underworld,” an additional reference to burrowing and aestivation in this species.

#### Holotype

UNSM 32104 (University of Nebraska State Museum) complete articulated skull with partial postcranium within aestivation burrow; from the Raney Farm locality (UNSM Loc. RH-104), Richardson Co., Nebraska, USA.

#### Referred specimen

UNSM 32108, complete partially disarticulated skull with partial pectoral girdle in aestivation burrow; from Raney Farm locality.

#### Locality and horizon

The type locality is the Raney Farm locality [16], Richardson Co., Nebraska, USA (UNSM Loc. RH-104). Fossils at the Raney Farm locality are found within the Eskridge Formation (Council Grove Group) within the lower P2 paleosol [23], a greyish-green vertisol with moderate pedogenesis. The P2 paleosol, and other paleosols within the Council Grove Group, represent subaerial exposure during lowstand intervals within broader Milankovitch-scale climatic oscillations. This unit lies well above the first occurrence of the Permian index fossil *Streptognathodus isolatus* in the Bennet Shale Member of the Red Eagle Limestone [24] but is below the first occurrence of the Sakmarian indicator species *Sweetognathus merrilli* in the Eiss Limestone member of the Bader Limestone [25], constraining the age to the Asselian (298.9–295.0 Ma).

#### Diagnosis

Oral margin unossified. No cosmine present on skull roof, cheek, and jaw. D absent. Q absent. Highly reduced circumorbital and cheek skeleton consisting of bones 5–8 only. Temporal series consists of a single compound bone (Y). Quadrate process of Y greater than 60% of the height of Y. Cranial sensory lines follow open canals in bone except on KLM and circumorbital series, where the sensory lines are enclosed in the bone and communicate with the surface via a series of two to three regular oval pits. Supratemporal commissure complete on posterior margin of B. EF with narrow deeply-keeled anterior process. Supraorbital sensory line canals form anterior commissure on anterior process of EF. Prearticular and entopterygoid tooth plates with four laterally-compressed denticle rows. Dermopalatine tooth plates subcircular and laterally compressed, with up to 14 individual denticles.

#### Differential diagnosis

Distinguishable from *Gnathorhiza* by the following suite of characters: anterior process of EF narrow without anterior flaring. Anterior process of EF deeply keeled ventrally. Parasphenoid diamond-shaped with short posterior stalk. Supraorbital sensory line communicates with surface via two to three regular oval pits. Prearticular and entopterygoid tooth plates with four laterally-compressed denticle rows.

### Description

The skull of *Persephonichthys chthonica* is nearly completely represented in the study material, which includes complete skull roof and cheek (Fig. 1,2), palate (Fig. 1F), lower jaw (Fig. 5A–C), opercular series (Fig. 1B,2B), partial quadrate, and partial braincase (Fig. 3,4). For comparative purposes, the alphanumeric nomenclatural system of Foster-Cooper [26] is used to refer to bones of the skull roof and cheek. Homologies with tetrapodomorph cranial elements [3] will be mentioned when relevant.

The type and referred specimens of *Persephonichthys chthonica* are small for a lungfish (skull length ∼2 cm). Both specimens are found in vertical tube-shaped sedimentary structures interpreted here and previously [16] as estivation burrows. The postcranium is incomplete in both specimens but suggests an animal with a total length of approximately 15 cm. A partial ribcage and series of thoracic neural spines is preserved in the type specimen. Ribs are long, slightly curved, and cylindrical in cross section. Small flakes of bone within the burrow structure appear to be scales, but lack fine surface detail. No vertebral centra are present. The presence of a completely ossified infraorbital series and of ossifications within the occiput and antorbital cartilages suggests that these specimens are not embryonic despite their small size, and are likely either adults or advanced juveniles.

The remarkably well-preserved skull roof and cheek are highly simplified in comparison with other Paleozoic species and approach the morphology seen in many early Mesozoic lungfishes. The skull roof consists of a median, lateral, and temporal series, and the cheek is comprised of a single suborbital row (Fig. 1,2). The median series is made up of an unpaired EF, paired C, and an unpaired B bone. There is no evidence of an ossified dermal mosaic overlying the ethmoid region, in contrast to the state observed for sagenodontids and all Devonian forms. The lateral series is comprised of bones KLM, J, and I. The temporal row consists of a single element, bone Y. The suborbital series consists of four robust and roughly quadrangular bones arranged in a single row (Fig. 2B). The mosaic of quadrangular cheek bones seen in sagenodontids and Devonian lungfishes is absent.

Some anatomy of the skull roof deserves further discussion. Ventrally, bone KLM sends a curving paddle-like process along the anterior orbital wall lateral to the ethmoid (Fig. 1B–D). This process is sutured to a short dorsal process on the dorsal surface of the entopterygoid, effectively providing rigid bony support of the palatal dentition against the skull roof. This antorbital bar is regularly described in Mesozoic and Cenozoic lungfishes, where it is typically identified as an ascending process of the entopterygoid and a descending process of the KLM, respectively [27]. Homology with the processus ascendens of the palatoquadrate has been suggested by some authors [28] but in *Persephonichthys* it appears that this structure is completely dermal and associated with the orbit rather than a visceral structure. We suggest referring to this structure as an antorbital bar comprised of antorbital processes of the KLM and entopterygoid to avoid future confusion.

Bone I sends an elongate paddle-like process posteroventrally, where it onlaps the exoccipitals (Fig. 2C). Similar processes have been observed in skull roofs of various Devonian [29] and post-Devonian [21] lungfishes. This structure has been called a salient process by some workers [21], and has been interpreted as a site of articulation with the shoulder girdle, but in three-dimensionally preserved specimens of *Persephonichthys* this process is very clearly associated with the exoccipitals, and no bony connection exists between the posterior skull roof and shoulder girdle. As bone I has been interpreted as a homolog of the tetrapodomorph postparietal [3], the lungfish condition seen in both crown lungfishes and *Persephonichthys* would appear to represent a parallel loss of the extrascapular series within dipnoans, a condition that is described as the evolution of a ‘neck’ in tetrapodomorphs. It is not impossible that posteriorly-directed processes off bone I (such as that seen in *Sagenodus* and many Devonian taxa) represent sites of articulation with the anocleithrum whereas posteroventrally-directed processes of bone I represent sites of articulation with the exoccipitals, but this raises two considerations. First, the anocleithrum primitively articulates with the extrascapular series (A, H, and Z) rather than the postparietals. Secondly, if this is the case, then the condition in *Sagenodus* and the condition in *Persephonichthys* represent distinct character states and interpretation of processes off bone I in other lungfishes must be careful to distinguish between these two states. Bone Y sends a long process ventrally. This process is thin, with a deep medial sulcus which would have accommodated the quadrate. Similar quadrate processes have been described in a number of Mesozoic and Cenozoic lungfishes [27] but the quadrate process of *Persephonichthys* is singular in its length, which exceeds three times the depth of the body of bone Y itself. The quadrate is weakly ossified in UNSM 32104, and little anatomy can be determined with confidence. A prominent articular condyle is present, however.

Lateral line canals are preserved as well-defined sulci in the skull roof, except on the KLM, where the supraorbital canal is more completely enclosed within the bone and communicates with the surface via a series of three wide oval fenestrae. The supraorbital, infraorbital, and supratemporal lateral line canals meet on the surface of bone Y. The supraorbital canal follows the arch of dorsal orbital margin on the KLM, exists the bone anteriorly, and then makes a strong median loop, reaching the midline of the anterior process of the EF. The infraorbital lateral line canal follows the infraorbital series anteriorly, sending off a branch towards the mandible within bone 5. The supratemporal lateral line canal traverses bone I and then follows the posterior margin of the skull roof towards the midline, where it forms the supraoccipital commissure.

The bony palate consists of a median parasphenoid, paired entopterygoids, and paired dermopalatines (Fig. 1F). The body of the parasphenoid is narrow and diamond-shaped and lies ventral to the entopterygoids. The parasphenoid lacks the broad, raised ‘lozenge’ that characterizes most Late Devonian and Carboniferous taxa typically considered relevant to modern lungfish origins. Paired fossae on the dorsal surface of the posterior half of the body of the parasphenoid represent impressions of the otic capsules (Fig. 3A). The posterior stalk of the parasphenoid comprises approximately a third of the total length of the element. An elongate sulcus is present on the dorsal surface of the posterior parasphenoid stalk there the palate would have accepted the notochord. A pair of dorsal processes lay lateral to this notochordal fossa, demarking the site of articulation for the cranial ribs (Fig. 3A,B). A shallow sulcus marks the ventral surface of the parasphenoid stalk along the path of the internal carotid artery and forks at the base of the body of the parasphenoid (Fig. 1F). The entopterygoids are broadly arched posteriorly. The entopterygoid tooth plates have only four tooth rows, each laterally compressed into a sharp cutting edge. The anterior tooth row is significantly deeper than the lateral tooth row, similar to the state described for *Orlovichthys limnatis* [30]. The dermopalatines are positioned lateral to the anterior tooth row of the entopterygoid, and consist of a rounded element with twelve to fourteen odontodes arranged in a single row (Fig. 1F). These elements have been interpreted as the vomers in a number of post-Devonian lungfishes [21], [27], [31] but the position lateral to the anterior tooth row rather than anterior to it would suggest that this element is homologous to the dermopalatines of Devonian lungfishes and not the vomer, which is an unpaired and often edentulous element in Devonian taxa.

The lower jaw is comprised of three bones, the splenial, angular, and prearticular (Fig. 5A–C). The splenial is a large rhomboidal element and makes up the anterior half of the external lower jaw, with a dorsoventrally-expanded contribution to the median symphysis. The dermal surface of the splenial is marked by dorsal and ventral branches of the mandibular lateral line canal as well as shallow pitting distributed across the anterior surface of the bone. The angular is the sole bone of the posterior lower jaw, and is elongate and wedge-shaped, with little surface morphology. The prearticular makes up the entire median surface of the lower jaw and supports the prearticular tooth plate. The prearticular tooth plate consists of four laterally-compressed tooth rows, the anteriormost nearly reaching the symphysis. Posteriorly, a small process is present in the coronoid region, possibly equivalent to the coronoid eminence seen in the diminutive gnathorhizid *Monongahela stenodonta* [32] but also various Devonian lungfishes [12], [20]. The adductor mandibulae would have inserted onto the lower jaw via a stout tendon at this coronoid eminence.

The most striking feature of these specimens is the presence of ossifications within the exoccipital (Fig. 3) and ethmoid (Fig. 4) regions, permitting description of this anatomy in a post-Devonian fossil lungfish for the first time. These ossifications consist of a combination of thin, probably perichondral, bone in some areas (e.g. the synotic tectum) and more completely ossified regions likely exhibiting some degree of endochondral ossification. It is not impossible that the braincase of these specimens was not completely ossified at the time of death, and that larger specimens may exhibit more complete ossification of the braincase, but the presence of more complete ossification, especially within the exoccipitals, would suggest that these specimens are postembryonic, even if not adult.

Exoccipitals are present and reasonably well-ossified in UNSM 32104, and are articulated weakly with the posterior stalk of the parasphenoid (Figure 3A–F). The occiput extends significantly behind the posterior margin of the skull roof. No medial contact is present between the exoccipitals; the synotic tectum is unossified (although paired projections from each exoccipital suggest that ossification of the synotic tectum was still in progress in this specimen), as is the basioccipital cartilage. A small process delineates the subtriangular foramen magnum from the larger notochordal pit, but does not meet medially to form an ossified shelf as in some Devonian taxa. Weakly-developed exoccipital condyles are present lateral to the notochordal pit. Anterior to the condyles, a shallow groove serving the jugular vein is evident on the lateral surface of the exoccipital and eventually terminates in a foramen that pierces the braincase just posteroventral to the otic capsule. A second foramen just anteroventral to the jugular foramen represents the entrance of the orbital artery. There are three foramina directly ventral to the jugular groove (Fig. 3C). The canals that exit at these foramina are simple and unbranching (Fig. 3E), and originate on the ventromedial surface of the occipital bone in the region of the hindbrain (3F). The anterior two canals clearly represent the paths of the glossopharyngeal and vagus nerves. The identity of the posterior canal is less certain. Although location and similarity to the canals for the glossopharyngeal and vagus nerves suggest that this canal did in fact carry a nerve as well, lungfishes do not have a true spinal accessory nerve or hypoglossal nerve [24]. However, many Late Devonian lungfishes add neural arches, centra, and ribs to the posterior end of the skull, entrapping the first spinal nerve. This ‘spino-occipital nerve’ has been identified in *Rhinodipterus kimberleyensis* [20] and *Griphognathus whitei* [33] in the same location of this foramen, and we consider these likely homologous. There is no evidence of a persistent otoccipital fissure as in *Soederberghia* and many other Devonian lungfishes, although this may represent a limitation of the CT resolution rather than a true absence of this feature. Anteriorly, the exoccipitals flare dorsally and laterally into a bulbous expansion that contributes to the base of the dorsolateral cristae, although the cristae themselves are incompletely ossified. No evidence is present of adlateral cristae.

The otic capsules are generally unossified, but some record of their presence is preserved. Large paired fossae are evident on the parasphenoid posterior to the lateral angle of the parasphenoid plate (Fig. 3A,B). Directly above each fossa is a single small, heavily-mineralized element identified here as an otolith (Fig. 3A). Otoliths are known from modern lungfishes [34] but their presence has not been documented within fossil lungfishes. There has been some debate on whether the plesiomorphic lungfish otolith complement is comprised solely of a single saccular statocone per otic capsule as in *Protopterus* [35], separate utricular and saccular statocones as described in *Neoceratodus* [34], or an aggregation of otocones [36], but the presence of a single otolith here, probably within the sacculus, in an extinct lungfish outside the crown group is consistent with the presence of a single saccular otolith in both actinistians and tetrapods, and lends further credence to the idea that a single otolith in the sacculus is plesiomorphic for sarcopterygians more broadly.

A portion of the left anterior neurocranium is ossified in UNSM 32108 (Fig. 4). This ossification represents a majority of the left ethmoid region between the nasal capsules and the anterior region of the orbit. Anteriorly the ossification flares anteroventrally to preserve the posterior wall of the nasal capsule (Fig. 4C,E). Posterior to this, a deep notch in the ventral margin of the element likely represents the foramina serving the profundus nerve (Fig. 4E). The medial surface of the element is marked by a longitudinal groove that likely served the olfactory nerve. The posterior margin of the bone is weakly notched, separating the facets for the attachment of the ventral and dorsal trabeculae, suggesting that the medial orbital wall was fenestrate as in juvenile *Neoceratodus forsteri* [37]. A region of unfinished bone along the dorsal margin of the element suggests that left and right ethmoid ossifications would have been bridged by cartilage, but were likely not connected by a broad nasal tectum as in *Neoceratodus* and many Devonian lungfishes [12], [28]. As with the anterior edge of the exoccipital bones, ossification of the ethmoid ends abruptly in both the orbital and nasal region.

Of the hyobranchial skeleton, only the ceratobranchial is ossified (Fig. 5D,E). The ceratohyal is an elongate element with an expanded proximal head and cylindrical shaft. The shaft makes up more than 60% of the length of the element and is only thinly ossified, likely perichondrally. A shallow notch is present on the ventral edge of the ceratohyal at the beginning of the expansion of the proximal head. A ridge on the medial surface delineates the origin of the interhyoideus muscle, and another on the ventrolateral surface delineates the insertion of the rectus cervicus. A tubercle on the lateral surface of the proximal head of the ceratohyal marks the location of the mandibulohyoid ligament.

The operculum and suboperculum are preserved at least in part in both specimens, but are better preserved in UNSM 32104 (Fig. 2B). The operculum is a large circular bone. A large ridge medially serves as the insertion of the opercular adductor musculature. A single sliver-like subopercular bone sits below the opercular.

The pectoral girdle is preserved in both UNSM 32104 and UNSM 32108 (Figure 1, 5F,G). The pectoral girdle is constructed of three separate ossified elements; the anocleithrum, the cleithrum, and the clavicle. The anocleithrum is preserved only in UNSM 32108, where it is a small, flat element. The cleithrum is incompletely preserved in both UNSM 32104 and UNSM 32108, but appears to be a narrow element without a significant branchial lamina. Supraglenoid and supracoracoid fossae are both present on the medial surface of the cleithrum. The clavicle is large and roughly triangular, flaring widely at the base. A medial lamina is present on the posterior surface of this element. These elements compare well with those described in *Gnathorhiza* cf. *G. serrata* [31], though the cleithrum and clavicle were reversed in this interpretation.

### Phylogenetic Analysis

**Figure 6:**
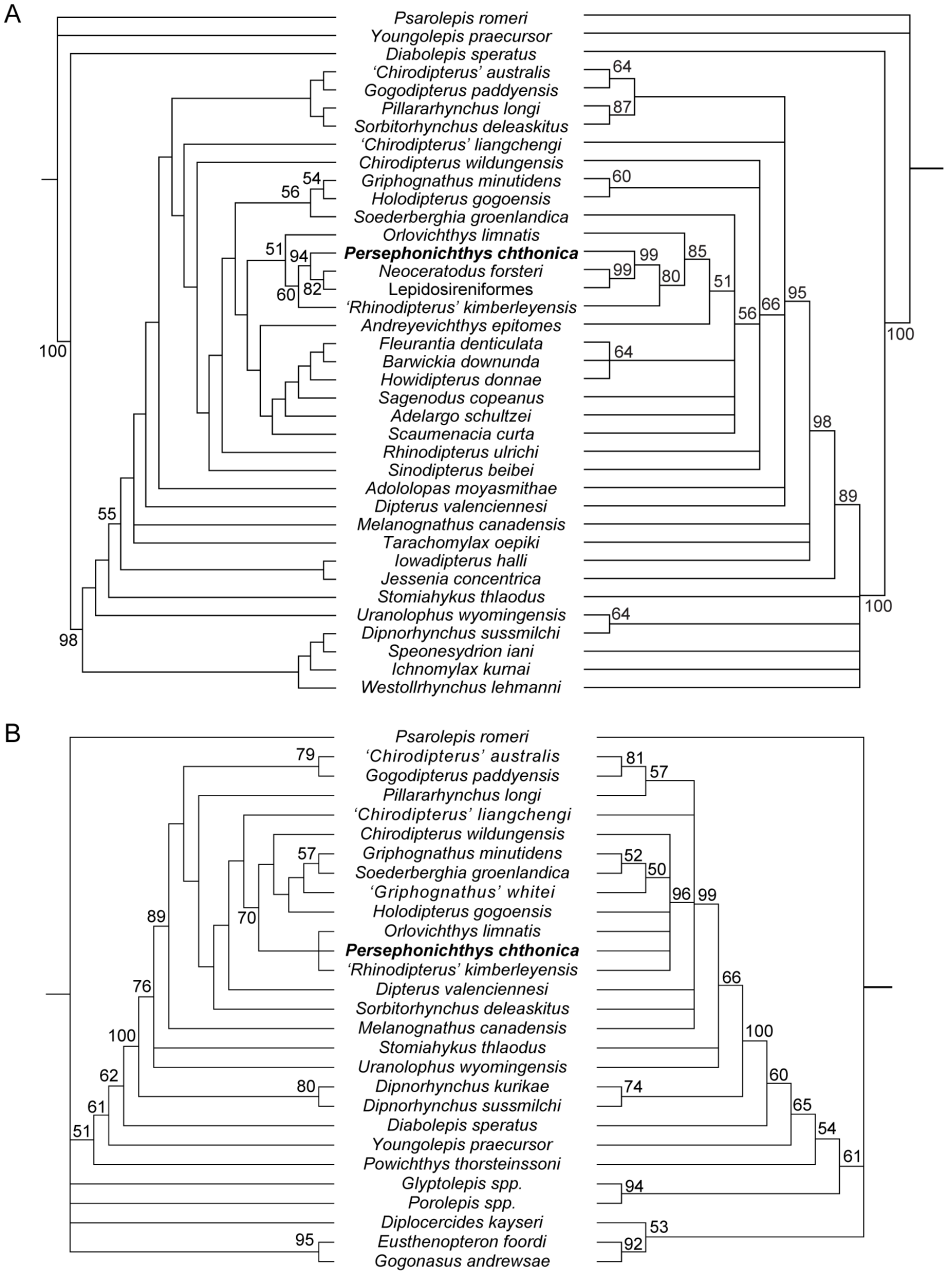
Phylogenetic relationships of *Persephonichthys cthonica* among selected Devonian lungfishes. **A**, phylogeny of *Persephonichthys chthonica* and selected Devonian lungfishes based on a comprehensive character sample, strict consensus of 2 maximum parsimony solutions left (TL: 505, CI: 0.3683, RCI: 0.2328) with bootstrap values provided for nodes with >50% bootstrap support, and Bayesian solution right (log likelihood of best state for cold chains −1742.09 and −1743.43) with clade credibility values provided for nodes with >50% clade credibility. **B**, phylogeny of *P. chthonica* and selected Devonian lungfishes based on a braincase-only character sample, strict consensus of 12 maximum parsimony solutions left (TL: 135, CI: 0.5333, RCI: 0.4232) with bootstrap values provided for nodes with >50% bootstrap support, and Bayesian solution left (log likelihood of best state for both cold chains −494.57) with clade credibility values provided for nodes with >50% clade credibility.

Our analysis of the neurocranial dataset [13] and the comprehensive character matrix [14] recover generally similar topologies (Fig. 6), a result that is not surprising, as the comprehensive matrix extensively samples characters identified by Friedman [13]. Maximum parsimony analysis of the comprehensive dataset (Fig. 6A) recovers *‘Rhinodipterus’ kimberleyensis* and *Orlovichthys limnatis* as successive Devonian outgroups to *Persephonichthys chthonica* and modern lungfishes. This topology is found in all most parsimonious trees (MPTs) and is supported by a weak majority of bootstrap replicates. The traditional placement of modern lungfishes is within the sister taxon of this clade, the ‘phaneropleurids’ (*Andreyevichthys*, *Adelargo*, *Barwickia*, *Scaumenacia*, *Howidipterus*), fleurantiids (*Fleurantia*), and sagenodontids (*Sagenodus*). This topology is largely replicated by the Bayesian analysis (Fig. 6A), which assigns posterior probabilities of 0.80 and 0.85 respectively to the identity of *‘R.’ kimberleyensis* and *O. limnatis* as successive outgroups to *P. chthonica* and modern lungfishes. Support for this topology is among the highest throughout the tree within both analyses. *Rhinodipterus* is found to be polyphyletic, with *Rhinodipterus ulrichi* occupying a position more basal than holodontids and rhynchodipterids. This analysis is not intended to serve as a test of monophyly of the genus *Rhinodipterus* but a revision of this genus is clearly necessary.

The neurocranial-only character matrix [13] produces similar results (Fig. 6B), but with less confidence. Maximum parsimony analysis of the neurocranial character matrix recovers a polytomy including *P. chthonica*, *O. limnatis*, and *‘R.’ kimberleyensis* in all MPTs, but without support in a majority of bootstrap replicates. Bayesian analysis of the neurocranial character matrix fails to recover majority support for phylogenetic resolution in the larger clade containing holodontids, rhynchodipterids, *O. limnatis*, *‘R.’ kimberleyensis*, and *P. chthonica*, however.

## Discussion

### Relationships of modern lungfishes

Our analysis of the relationships of *Persephonichthys chthonica*, and thus the relationships of crown lungfishes suggests some intriguing possibilities. Most important of these is the possibility that the diversity of ‘phaneropleurids,’ ‘fleurantiids,’ and ‘sagenodontids,’ from the Late Devonian and Late Paleozoic are less closely related to *Persephonichthys* and crown lungfishes than some Late Devonian forms, namely the ‘dipterids’ *Orlovichthys limnatis* and *‘Rhinodipterus’ kimberleyensis*. In our analysis of the braincase character matrix and of the comprehensive matrix, we recover a close relationship between *P. chthonica* and the Famennian lungfishes *‘Rhinodipterus’ kimberleyensis* and *Orlovichthys* to the exclusion of the ‘rhynchodipterids’ *Soederberghia groenlandica*, *Griphognathus minutidens*, and *‘Griphognathus’ whitei*. This result is present in all most-parsimonious trees for both matrices, and is robust to bootstrapping in the comprehensive matrix.

Although the matrix used in this analysis was designed specifically to investigate relationships between Devonian lungfishes [14], we are confident in our identification of *Persephonichthys* as the closest Paleozoic relative of the lungfish crown due to a number of crown synapomorphies readily identifiable in the skull of *Persephonichthys* that are absent in all other Paleozoic lungfishes, with the possible exception of *Gnathorhiza*. Most obvious of these synapomorphies is the antorbital bar formed from processes of the skull roof and entopterygoid. This structure is present in *Neoceratodus* and all lepidosirenids, and has been described in the Mesozoic lungfishes *Arganodus atlanticus* [27] and *Ferganaceratodus* [38], and the Cenozoic lungfish *Mioceratodus gregoryi* [27]. The cranial lateral line canals are housed within sulci on the surface of the bone, rather than within canals enclosed by bone. The entire extrascapular series is completely lost. The supraorbital bones of the circumorbital series are lost. A cranial rib is present and articulates with a facet on the parasphenoid. A single median compound bone is present in the anterior skull roof in the region of the E and F bones. A mosaic of small elements is completely absent, as is an ossified oral margin.

One of the most striking characteristics of our phylogeny is the pervasive non-monophyly of post-Devonian lungfishes. A clade comprising most if not all post-Devonian lungfishes has traditionally been recovered due to a number of shared characters, including the loss of cosmine, reduction in the complexity of the median fin structure and supports, loss of a heavily ossified oral margin, and reduction of endochondral ossification throughout the skeleton. Our results suggest that this suite of characters probably evolved in parallel in at least one lineage in the Late Devonian (*Barwickia*+*Howidipterus*+sagenodontids) and a second lineage in the late Paleozoic (*Persephonichthys*+crown lungfishes), and may reflect broader patterns of convergence in ecology and function. Although it is uncertain how salt-tolerant Late Paleozoic lungfishes were [21], [39], what is clear is that after the Hangenberg extinctions, Late Paleozoic lungfishes vacated typical marine niches, especially those associated with feeding on reef-building marine invertebrates, and repeatedly colonized freshwater or estuarine environments. Much of the convergence within Late Devonian and Late Paleozoic lungfishes more generally involves reduction of ossification and biomineralization, including loss of cosmine, reduction of endochondral ossification, and reduction of the dermal skull. Reduction of ossification with colonization of freshwater ecosystems is pervasive in some modern fishes [40], [41], [42] and may be a response to reduced predation risk from gape-limited gnathostome predators [40], to limited access to calcium [40], [42] or a combination of these factors [40], [41] It is possible that patterns of bone reduction among Late Devonian and Late Paleozoic lungfishes (and Late Devonian and Late Paleozoic osteichthyans more generally) may be, in part, a reflection of the differences in selective regime between marine and freshwater ecosystems, a possibility which should be investigated further.

Furthermore, this calls into question the conclusions of some previous authors [2], [10] that the rate of lungfish evolution has been essentially negligible after the end of the Devonian. *Persephonichthys* demonstrates numerous crown lungfish synapomorphies, such as the presence of an antorbital wall formed by processes of the pterygoid and bone KLM, absent in other Paleozoic forms, but the braincase of *Persephonichthys* differs markedly from both extant lepidosirenids and *Neoceratodus* in being suspended beneath the skull roof via cristae allowing for insertion of the epaxial musculature between the skull roof and braincase as in Devonian forms, in the presence of an extended occiput enclosing a spinal nerve, and in the presence of separate foramina in the occipital region serving the glossopharyngeal nerve, vagus nerve, and jugular vein separately as opposed to a single metotic fissure as in lepidosireniforms [28]. In addition, the lepidosirenid braincase demonstrates major reductions of the trabecular cartilages and dorsal braincase, with significant reorganization of the suspensorium. The rate of evolution of these structures is difficult to deduce due to the sparse fossil record of lungfish braincases, but the combination of more general plesiomorphic lungfish anatomy and autapomorphies unique to *Persephonichthys* suggests more substantial morphological evolution within post-Devonian lungfishes than previously thought. As opposed to representing true evolutionary stasis, this suggests that rate heterogeneity identified by previous workers [2], [10] may instead be a function of wholesale missing data in the post-Devonian lungfish fossil record. The vast majority of post-Devonian lungfishes are represented solely by isolated tooth plates, with most remaining post-Devonian taxa represented by the relatively character-poor skull roof [8], [27], [38]. As an extreme example, a recent phylogenetic analysis of post-Devonian lungfishes [38] identified only 14 characters (primarily gross anatomy and skull roof), whereas the analysis of Devonian lungfish relationships by Friedman [13] identified 70 characters from the braincase alone. This discrepancy between the relative character richness of anatomical regions available for Devonian taxa and for later taxa presents a likely confounding factor in studies of evolutionary rates in lungfishes. Without additional exceptionally-preserved fossils of post-Devonian lungfishes, hypotheses of changing evolutionary rates within the lineage are difficult to test. Fortunately, the unexpected presence of ossified braincase in *Persephonichthys chthonica* suggests that remains of the neurocranium may be present in other post-Devonian lungfish fossils. Although preservation of *P. chthonica* is exceptional, this does not appear to be the result of unique taphonomic conditions. The identification of braincase in *P. chthonica* comes from application of new imaging modalities to the study of these specimens rather than exceptional depositional conditions. Use of micro-CT and synchrotron scanning to restudy three-dimensionally preserved lungfish skulls, including well-studied fossils like *Ceratodus sturii* and enigmatic fossils such as *Namatozodia*, may reveal braincases for these taxa as well, making a robust and credible phylogeny of at least a few post-Devonian lungfishes achievable.

### Buccal pumping in lungfishes

The idea that the origin of cranial ribs within Late Paleozoic lungfishes indicates the origin of air-breathing within this lineage has been around for some time [43], but has recently been revisited [20], [44] in their interpretation of the skull of *‘Rhinodipterus’ kimberlyensis*. Their argument refers primarily to the functional morphology of *Lepidosiren paradoxa*, in which some action of the M. rectus cervicis (which originates in part along the cranial rib in modern lungfishes) has been identified during the inspiration phase of air-breathing [4]. Clement [20] provides a more extended list of characters interpreted as indicating air-breathing capacity, including an elongate parasphenoid, curved thoracic ribs, a loose attachment between the anocleithrum and skull, and a gap between the prearticular tooth plates. The elongate parasphenoid is interpreted by Clement as representing an enlargement of the buccal cavity, the curved thoracic ribs are interpreted as forming a space for larger lungs, the gap between the prearticular tooth plates is interpreted as serving a stopgap function while pumping air into the lungs, and the loose attachment between the anocleithrum and skull is interpreted as an adaptation for expansion of the buccal cavity during inspiration. Clement [20] acknowledges a role of these characteristics in buccal pumping more generally, but argues that extrapolation of these characteristics in *‘Rhinodipterus’ kimberleyensis* may indicate increased reliance on air-gulping.

One of these lines of evidence is dubious (the elongation of the parasphenoid crownward on the phylogeny likely represents an expansion of the posterior braincase, both via the expansion of the otic capsule and the addition of cranial centra to the back of the skull, and an anterior displacement of the tooth plates for prey capture), whereas others (dissociation of the pectoral girdle from the skull and the gap between the tooth plates) are suggestive of more effective buccal pumping as claimed by Clement [20], although not necessarily air-breathing. It is worth pointing out that a gap between the prearticular tooth plates is seen in a variety of Devonian lungfishes [12], and that, where present, it appears to accept the anterior tooth rows of the entopterygoid tooth plates during occlusion.

The use of cranial ribs as indicators of fossil lungfish behavior is also somewhat difficult. Cranial ribs are frequently identified in Late Paleozoic lungfishes, including the Carboniferous-Permian taxon *Sagenodus* [21], but cranial ribs are found throughout dipnoans from the Middle Devonian onwards. Two pairs of thickened ribs have been identified in the occipital region of *Dipterus valenciennesi* and interpreted as cranial ribs [45]. *Dipterus* is a Middle Devonian (Givetian) lungfish typically considered further from the crown than *Rhinodipterus* or any Late Paleozoic form. A similar interpretation has been made of the occiput of *Soederberghia simpsoni* [46], and has been used to unite *Soederberghia* and *Rhynchodipterus*, and to generally support the monophyly of a rhynchodipterid clade. Friedman [47] has challenged this interpretation of *Rhynchodipterus*, however, pointing out that the described ribs in *S. simpsoni* resemble the two pairs of thickened anterior thoracic ribs found in *Griphognathus* and may not articulate with the cranium at all. We suggest a third alternative: the thickened anterior thoracic ribs may be homologous with the cranial ribs, and serve as the origin of portions of the M. rectus cervicus as well, and that articulation of these ribs with the occiput, or lack thereof, may reflect the fusion of post-occipital centra with the braincase. Cranial centra are added and lost throughout early lungfish phylogeny [33], and it is possible if not likely that other axial components, such as ribs, might be incorporated into or lost from the posterior neurocranium as these centra are added or lost. Such an interpretation, if demonstrated to be correct, has three primary implications. The first is that presence or absence of cranial ribs becomes less important for functional concerns than the presence or absence of thickened anterior thoracic ribs. Functionally, what matters is that a head of the M. rectus cervicus originates on the axial skeleton, permitting rotation as well as abduction of the ceratohyal during hyoid abduction and mandibular depression. As this character seems to be widely distributed among dipnoans, the utility of this character in inferring air-breathing adaptation may be overstated, and probably reflects adaptation for buccal pumping more generally. Secondly, this suggests that presence/absence of cranial centra and presence/absence of cranial ribs are not independent characters, and must be conceptualized together in future phylogenetic analyses. Third, this suggests that isolated ribs identified as ‘cranial ribs’ (e.g. the cranial rib of *Sagenodus*) may not represent true cranial ribs at all, and that identification of a true cranial rib requires first the identification of articular facets for the cranial ribs on either the occiput or parasphenoid. Such structures are rare among Paleozoic lungfishes, present in *‘Rhinodipterus’ kimberleysis* [20], [44], where they articulate with the occipitals, the gnathorhizid *Gnathorhiza* [31], and now *Persephonichthys chthonica*, where they occur on the parasphenoid, as in post-Devonian lungfishes [27].

More generally, the evolution of the cranial ribs in Devonian lungfishes reflects a more general transition from obligate durophagy in early dipnoans towards a more catholic diet in extant lungfishes. Diet in modern lungfishes incorporates a significant amount of active prey, both benthic and pelagic. *Neoceratodus forsteri* has been shown to feed on fish, arthropods, annelids, and molluscs [48], and *Protopterus* has been found to be primarily piscivorous [49]. Capture of more active prey requires a more dynamic feeding apparatus. This is accomplished in lungfishes via a highly derived form of buccal pumping. Buccopharyngeal pumping is likely basal for osteichthyans, and the feeding cycle of plesiomorphic actinopterygians, such as *Polypterus*, may present a reasonable analog for feeding in early lungfishes. The feeding cycle of *Polypterus* consists of five main phases: (1) gape, (2) hyoid depression, (3) opercular abduction, (4) mandibular adduction, and (5) hyoid adduction [50]. Of these, gape and hyoid depression are most important in determining the speed at which suction is produced and the force of that suction, whereas subsequent phases are important for prey manipulation and processing. Gape itself consists of two events: depression of the mandible and elevation of the neurocranium. Mandibular depression is linked with hyoid depression via the mandibulohyoid ligament, with the M. sternohyoideus ( = M. rectus cervicus) as the primary agonist. Neurocranial elevation is accomplished via action of the epaxial muscles, which insert along the occiput. In plesiomorphic actinopterygians, neurocranial elevation is restricted due to close integration between the bones of the skull roof and dermal shoulder girdle; and is lost entirely in teleosts in favor of a more mobile premaxilla supported by linkages within the dermal skeleton [51]. Although it has been suggested that the intracranial joint of sarcopterygians may have assisted in widening gape and contributing to bite force generation, recent work has shown that the intracranial joint in the modern coelacanth *Latimeria* is essentially immobilized by substantial basicranial muscles and may serve to dissipate force experienced during biting instead [52].

**Figure 7:**
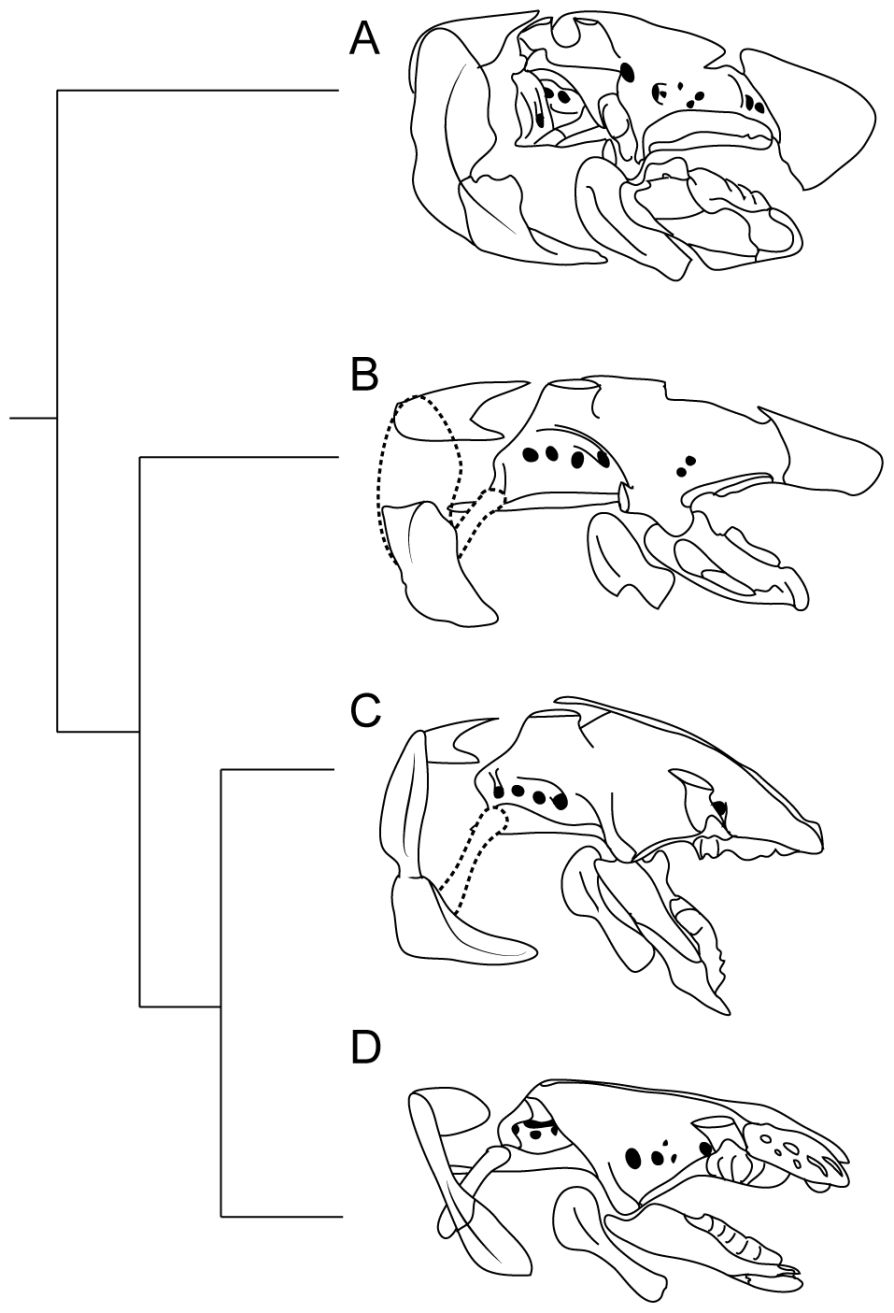
Summary cladogram illustrating the evolution of the jaw suspension and neurocranium in Dipnomorpha. **A**, *‘Chirodipterus’ australis*, after Miles [12]; **B**, *‘Rhinodipterus’ kimberleyensis*, after Clement [20]; **C**, *Persephonichthys chthonica*, composite of UNSM 32104 and UNSM 32108; **D**, *Neoceratodus forsteri*, after Gunther [55].

Early lungfishes deviate from this system in a number of ways that have significant implications for function. Most obvious is the fusion of the anterior braincase, posterior braincase, and palatoquadrate into a single element and the close integration of the palate, especially the entopterygoids, with the braincase, forming a robust and immobile support for the entopterygoid tooth plates [3], [12], resulting in a fully autostylic skull. The hyoid arch is also relatively immobile, with a broad articulation between the ceratohyal and braincase, and an abbreviated ceratohyal with limited attachment sites for the rectus cervicus musculature. In addition, the pectoral girdle is well-integrated with the posterior skull via the extrascapular series (bones A, H, and X), which articulate suturally with the pectoral girdle via the anocleithrum. The conical dentition of dipnoan outgroups is also replaced by a variety of derived palatal tooth arcades, generally involving a combination of denticles as well as extensive extradenteonal dentine [5]. In some taxa, this dentition forms a robust crushing surface, although in other taxa, the distribution of palatal denticles has been inferred to serve as a rasp [47]. The skull of most Devonian lungfishes would have been capable of exerting and resisting large bite forces, likely from crushing marine invertebrates with mineralized exoskeletons, but at the expense of gape and buccal suction. A general trend toward this particular construction of the jaw and suspensorium and articulation of cranial rib is illustrated by the morphology of *‘Chirodipterus’ australis* (Figure 7A).

Modern lungfishes (Figure 7D) and *Persephonichthys* (Figure 7C) modified this system substantially and thus likely achieved a more dynamic feeding cycle that increased the speed of gape and hyoid depression. The aforementioned expansion of the M. rectus cervicus onto the cranial ribs is one such modification, but there are a number of others, including the elongation of the ceratohyals, loss of the ossified oral margin, anterior displacement of the palatal dentition, and evolution of an inclined rather than vertical suspensorium, all characteristics present in *Persephonicthys* as well as modern lungfishes, but absent in more basal forms such as *Sagenodus*. Most notably, however, extrascapulars (bones A, H, and Z) have been completely lost in *Persephonichthys* and no anocleithral facet is present on the occiput, precluding a tight articulation between the anocleithrum and skull roof and permitting a greater range of neurocranial elevation via the epaxial musculature. Interestingly, the suspension of the braincase beneath the skull roof via the dorsolateral cristae in *Persephonichthys* suggests insertion of the epaxial musculature within a chamber between the skull roof and braincase as in Devonian lungfishes and sarcopterygians more generally, rather than insertion of these muscles along the dorsal surface of the posterior skull roof as in modern lungfishes. This suggests substantial reconfiguration of the occipital region relatively late in lungfish evolution.

The dissociation of the pectoral girdle from the posterior skull in *Persephonichthys* as an adaptation towards more effective buccal pumping represents a significant change in function within Late Paleozoic lungfishes and suggests intriguing parallels with late stem-tetrapods. A similar dissociation between the skull and pectoral girdle is recorded among elpistostegalid-grade stem-tetrapods, and has been described as the “origin of the tetrapod neck” [53]. It has been suggested that the presence of a ‘neck’ in stem-tetrapods implies lateral mobility of the head with respect to the body axis, and this has been invoked as a line of evidence in support of terrestrial prey capture in the ‘elpistostegalid’ *Tiktaalik roseae*. The presence of a similar dissociation between the skull and pectoral girdle within the lungfish stem suggests an alternate possibility: the loss of a bony connection between the skull and pectoral girdle in stem-tetrapodamorphs may represent an adaptation for neurocranial elevation and thus towards more effective buccal pumping in this taxon as well. Given that a broad, flattened skull (as is seen in ‘panderichthyid’ and ‘elpistostegalid’ stem-tetrapods) has also been shown to represent an adaptation towards suction feeding in the giant salamander *Andrias* [54], it may be fruitful to reconsider the origins of key tetrapod cranial characteristics as adaptations for aquatic, rather than terrestrial, prey capture.

## Conclusions

*Persephonichthys chthonica* is an exceptionally-preserved lungfish from the Early Permian of Nebraska, USA. Study of skulls of *P. chthonica* using micro-CT has provided the first evidence of fossilized neurocranium in a non-Devonian lungfish, and robustly supports a heterodox interpretation of lungfish phylogeny and modern lungfish origins. Monophyly of post-Devonian lungfishes is not supported. *Persephonichthys chthonica* is found to occupy a transitional position between the dipterid-grade lungfishes *Orlovichthys limnatis* and *‘Rhinodipterus’ kimberleyensis* and modern ceratodontiform lungfishes, and exhibits a transitional morphology of the suspensorium, hyobranchial skeleton, pectoral girdle, and occiput. We suggest that the origin of modern lungfishes was characterized by evolution of the feeding mechanism away from the static durophagy of Devonian dipterid-grade forms and towards a more dynamic buccal pump, permitting capture of more active prey. This may reflect a more general trend towards a dynamic subaqueous feeding cycle in other Paleozoic osteichthyan lineages, including stem-tetrapods.

## Supporting Information

Appendix S1 **Comprehensive character matrix.**
(NEX)

Appendix S2 **Neurocranium-only character matrix.**
(NEX)
